# Clear Cell Renal Cell Carcinoma: Local Recurrence and Bilateral Adrenal Metastases - A Case Report

**DOI:** 10.5334/jbsr.2772

**Published:** 2022-05-06

**Authors:** Eliza-Maria Popa, Rosana Manea, Roxana-Elena Birla-Coroiu

**Affiliations:** 1 Clinical and Emergency Hospital of Brasov, RO; 2 Transilvania University of Brasov, RO

**Keywords:** clear cell renal cell carcinoma, local recurrence, adrenal metastases, renal tumor, computed tomography

## Abstract

**Introduction:**

Clear cell renal cell carcinoma is the most frequent type of renal cell carcinoma, which is often diagnosed incidentally in an advanced stage.

**Case History:**

We present the case of a 49-year-old man who presented to the emergency department with no specific symptoms. After computed tomography (CT) evaluation, the suspicion was raised of a left renal tumour. The aim of this case study is to underline the importance of rapid diagnosis and further investigation of clear cell renal cell carcinoma and the severity of this type of cancer.

**Conclusions:**

Clear cell renal cell carcinoma has no specific symptoms. For the complete diagnosis and further monitoring, the use of CT is necessary.

**Teaching Point:**

Clear cell renal cell carcinoma treated with partial nephrectomy can relapse near the surgical scar and progress with metachronous bilateral adrenal metastases, especially when close follow-up is not performed due to the pandemic situation.

## Introduction

Clear cell renal cell carcinoma is the most frequent type of renal cell carcinoma, which, in the great majority of cases has an asymptomatic clinical behavior [1]. For this reason, often, renal masses are diagnosed in an advanced stage during investigations for other purposes [1]. The most frequent sign that may occur at the laboratory evaluation is microscopic haematuria. A CT scan is performed to determine the cause because this can appear from other reasons like nephrocalcinosis [2].

The purpose of this case study is to determine the value of CT scan in the diagnosis and post-treatment monitoring clear cell renal cell carcinoma.

## Case History

A 49-year-old man, with no personal history and without treatment, was admitted to the hospital with nausea and persistent epigastric abdominal pain for two days for which he self-administered an antispastic drug. The clinical examination and the blood tests from the first evaluation were normal.

**Figure 1:**
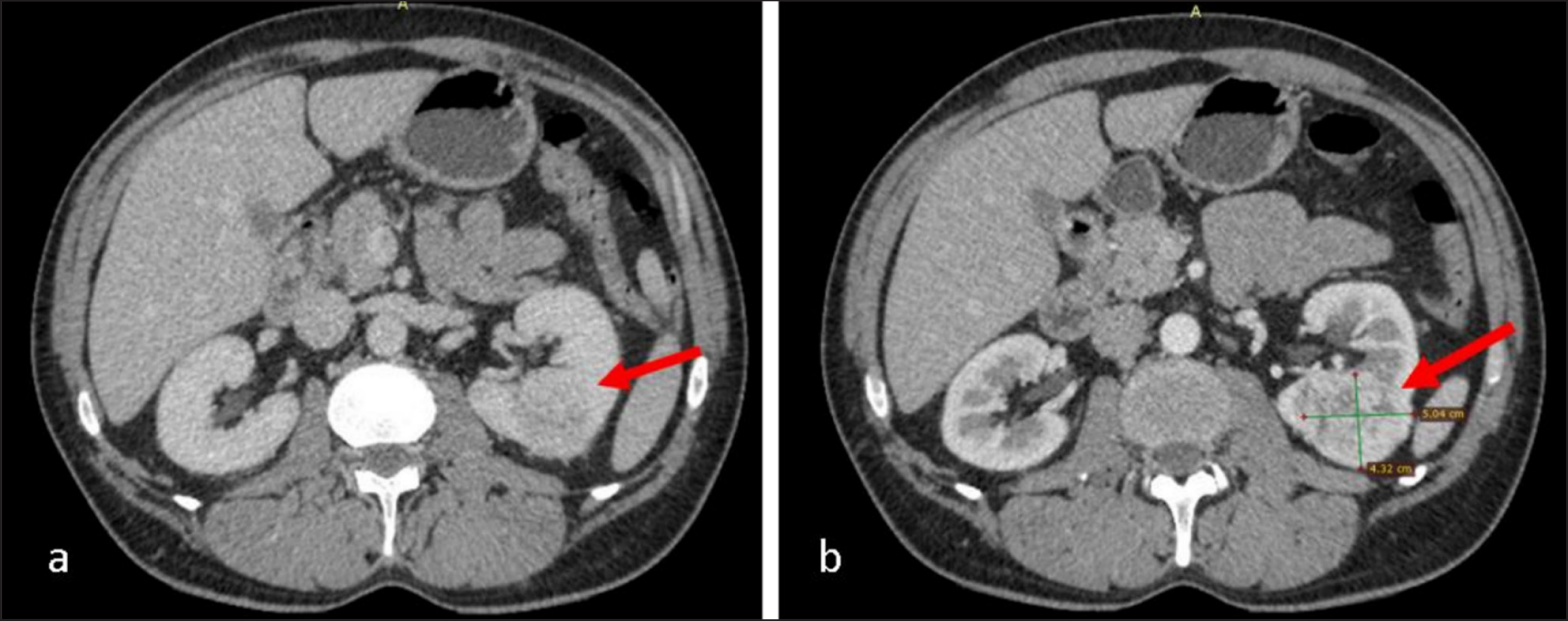
CT scan with contrast administration (CA): **a.** heterogeneous exophytic nodular lesion in the lower pole of the left kidney with small bulging into sinus, measuring 4.3/5/6 cm (AP/T/CC); **b.** the lesion enhances at periphery and has an irregular necrotic centre.

An abdominal CT scan with contrast administration was performed (***Figure 1***), and the appearance raised the suspicion of a left renal tumour.

After one-month, partial nephrectomy is performed, and the left renal lesion is totally removed. The clinical evolution after the surgery was favourable. The histopathological exam revealed clear cell renal carcinoma, and the patient went home with the recommendation to have another CT-scan investigation after six months.

Because of the pandemic situation, the patient did not follow any treatment and have postponed the scheduled investigation.

**Figure 2:**
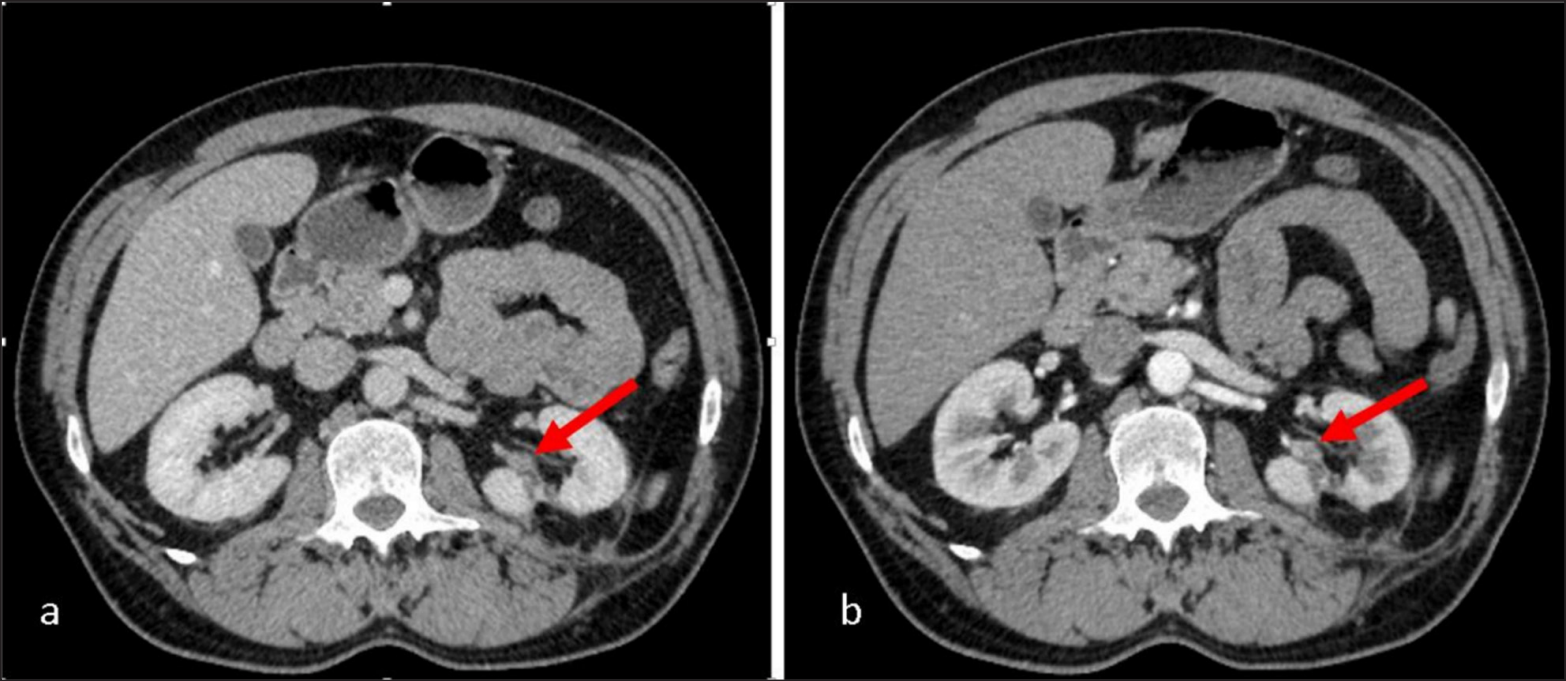
CT scan with CA: **a.** heterogeneous nodular lesion in the sinus of left remnant kidney; **b.** the lesion enhances at periphery and has necrotic centre.

**Figure 3:**
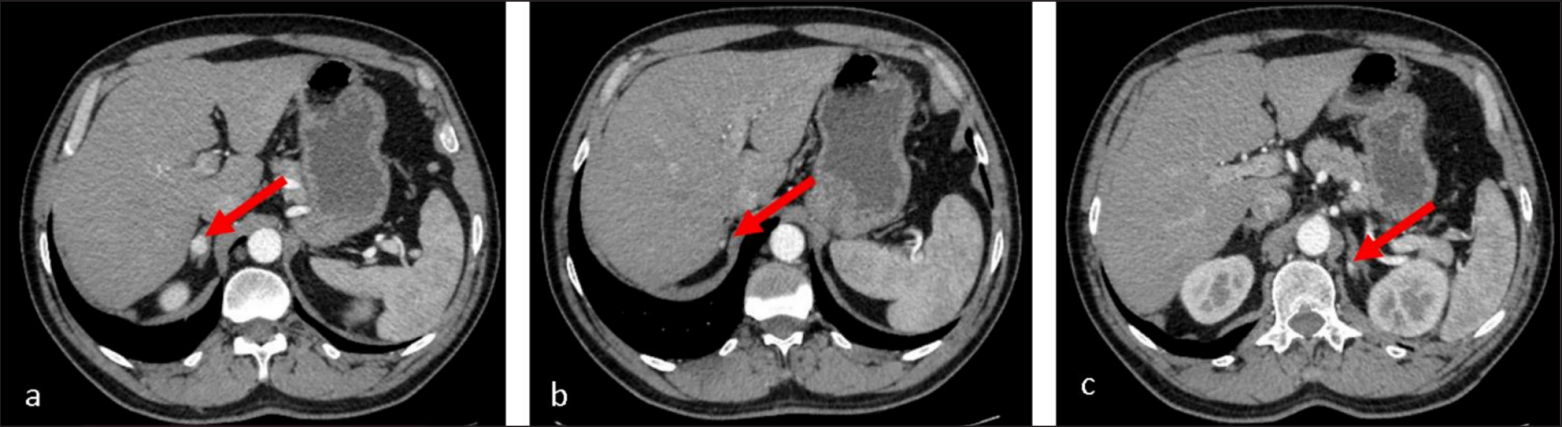
Arterial phase CT scan: **a, b.** two, nodular, enhancing solid lesions in the right adrenal gland; **c.** a solitary, nodular, enhancing solid lesion in the left adrenal gland.

Another control CT scan with contrast administration was performed after one year and four months which revealed the appearance of a new left sinusal renal nodular lesion (***Figure 2***) and bilateral adrenal nodular lesions, suggestive for adrenal metastases (***Figure 3***).

For the recently discovered lesions, the patient was recommended for chemotherapy for three months. After six months, a control CT scan revealed an evolutive aspect of the left sinusal renal lesion, with increased size with 25% and increased size of the bilateral adrenal lesions with 20%.

Considering the progressive imaging aspect and the poor prognosis, the medical team decide that left nephrectomy followed by adjuvant immunotherapy and chemotherapy is the best decision for the patient treatment.

## Comment

Over 90% of renal cancers are represented by renal cell carcinoma. Seventy percent of these are represented by clear cell renal cell carcinoma [3]. We presented the case of a patient with left-sided clear cell renal cell carcinoma who was incidentally diagnosed and for whom it was performed partial nephrectomy. After one year and four months, another abdominal CT investigation found a local recurrence of the kidney tumour and simultaneous bilateral adrenal metastases.

According to the literature, in most cases the patients have no symptoms that suggest this disease. Approximately 30% of patients come to the hospital with symptoms related to local advanced or metastatic disease [1,4].

Among the most frequent metastatic sites of kidney cancer is ipsilateral adrenal gland [4], and it can appear synchronous between 2–10% of cases or metachronous, which is a rare event [5].

Simultaneous bilateral adrenal metastases were found only in less than 1% of patients; there are approximately 20 cases described in literature [6].

## Conclusions

We presented the case of a 49-year-old man who was diagnosed incidentally with left renal mass after an abdominal CT scan. The histopathological result was clear cell renal cell carcinoma. The evolution of the patient was unfavourable and after a period local recurrence and simultaneous bilateral adrenal metastases were found.

The prognosis of clear cell renal cell carcinoma is poor, with high mortality; therefore a rapid diagnosis and treatment are required.
